# Low Efficiency of Homology-Independent Targeted Integration for CRISPR/Cas9 Correction in the Vicinity of the *SLC26A4* c.919-2A>G Variant

**DOI:** 10.3390/ijms26114980

**Published:** 2025-05-22

**Authors:** Chang-Han Ho, Cheng-Yu Tsai, Chi-Chieh Chang, Chin-Ju Hu, Cheng-Yen Huang, Ying-Chang Lu, Pei-Hsuan Lin, Chin-Hsien Lin, Han-I Lin, Chih-Hsin OuYang, Chuan-Jen Hsu, Tien-Chen Liu, You-Tzung Chen, Yen-Hui Chan, Yen-Fu Cheng, Chen-Chi Wu

**Affiliations:** 1 Department of Otolaryngology-Head and Neck Surgery, National Taiwan University Hospital, Taipei 100225, Taiwan; 2 Graduate Institute of Medical Genomics and Proteomics, National Taiwan University College of Medicine, Taipei 100233, Taiwan; 3 Program in Speech and Hearing Biosciences and Technology, Harvard Medical School, Boston, MA 02115, USA; 4 Gene Knockout/in Cell Line Modeling Core, Human Disease Modeling Center, First Core Laboratory, Branch Office of Research and Development, National Taiwan University College of Medicine, Taipei 100233, Taiwan; 5 Department of Neurology, National Taiwan University Hospital, Taipei 100225, Taiwan; 6 Department of Otolaryngology-Head and Neck Surgery, Taichung Tzu Chi Hospital, Buddhist Tzu Chi Medical Foundation, Taichung 427, Taiwan; 7 Department of Medical Research, Taipei Veterans General Hospital, Taipei 112201, Taiwan; 8 Department of Otolaryngology-Head and Neck Surgery, Taipei Veterans General Hospital, Taipei 112201, Taiwan; 9 School of Medicine, National Yang-Ming Chiao-Tung University, Taipei 112304, Taiwan; 10 Department of Speech Language Pathology and Audiology, National Taipei University of Nursing Health Sciences, Taipei 112303, Taiwan; 11 Department of Medical Research, National Taiwan University Hospital Hsin-Chu Branch, Hsinchu 302058, Taiwan

**Keywords:** CRISPR/Cas9, homology-independent targeted integration, *SLC26A4*, Pendred syndrome, hearing loss, gene editing

## Abstract

Recessive variants of *SLC26A4* are a common cause of hereditary hearing impairment and are responsible for non-syndromic enlarged vestibular aqueducts and Pendred syndrome. Patients with bi-allelic *SLC26A4* variants often suffer from fluctuating hearing loss and recurrent vertigo, ultimately leading to severe to profound hearing impairment. However, there are currently no satisfactory prevention or treatment options for this condition. The CRISPR/Cas9 genome-editing technique is a well-known tool for correcting point mutations or manipulating genes and shows potential therapeutic applications for hereditary disorders. In this study, we used the homology-independent targeted integration (HITI) strategy to correct the *SLC26A4* c.919-2A>G variant, the most common *SLC26A4* variant in the Han Chinese population. Next-generation sequencing was performed to evaluate the editing efficiency of the HITI strategy. The results showed that only 0.15% of the reads successfully exhibited HITI integration, indicating that the c.919-2 region may not be a suitable region for HITI selection. This suggests that other site selection or insertion strategies may be needed to improve the efficiency of correcting the *SLC26A4* c.919-2A>G variant. This experience may serve as a valuable reference for other researchers considering CRISPR target design in this region.

## 1. Introduction

Recessive variants in *SLC26A4* (MIM #605,646) represent the second most common cause of hereditary hearing impairment (HHI) after recessive *GJB2* variants occurring in 15–20% of patients with HHI [1,2]. Pathogenic *SLC26A4* variants have been linked to non-syndromic deafness DFNB4 (MIM #600,791) and Pendred syndrome (PS, MIM #274,600), both of which involve an enlarged vestibular aqueduct and progressive/fluctuating sensorineural hearing impairment (SNHI). Patients with PSalso exhibit thyromegaly in addition to inner ear manifestations [3,4]. Since most patients with non-syndromic DFNB4 or PSare not born with congenitally profound SNHI, there seems to be a therapeutic time window during which gene therapy can be applied to intervene or halt the deterioration of SNHI [5].

*SLC26A4* is located on chromosome 7q22.3 and consists of 21 exons [6]. The *SLC26A4* transcript (~57 kb, NM_000441.2) encodes the pendrin protein, which transports iodide, chloride, and bicarbonate in the inner ear, thyroid, kidney, salivary ducts, respiratory tract, liver, and heart [7,8,9,10,11,12]. Dysfunctional mutant pendrin has been shown to predispose cochlear sensory epithelial cells to aggregation and degeneration, thereby reducing cellular tolerance to oxidative stress [7].

To date, more than 600 pathogenic and likely pathogenic variants of *SLC26A4* have been documented in the Deafness Variation Database (version 9, https://deafnessvariationdatabase.org/, accessed on 15 May 2025) [13]). The predominant variants vary between populations [14]. Among them, the c.919-2A>G variant is highly prevalent in East Asian populations, including Han Chinese [15], Han Taiwanese [16,17], Japanese [18], Korean [19], and Mongolian [20]. The *SLC26A4* c.919-2A>G substitution at the splice site of intron 7 causes the skipping of exon 8, resulting in the production of a prematurely truncated protein in which exons 7 and 9 are joined [21].

The CRISPR/Cas9 system has recently been widely used as a powerful gene-editing tool in modern transgenic studies [22]. The principle of this technique is to introduce site-specific double-strand breaks (DSBs) using single-guide RNA (sgRNA) targeting and the Cas9 nuclease. When the protospacer and PAM motif of the sgRNA recognize the target sequence, the Cas9 nuclease unwind the genomic DNA duplex and cleaves the sequence 3 base pairs (bp) upstream of the PAM site, generating a predominantly blunt-end DSB. In the presence of donor DNA with two flanking homology arms, DSBs can be repaired via the precise homology-directed repair (HDR) pathway, or the DNA ends can be rejoined via the error-prone non-homologous end joining (NHEJ) mechanism [23].

However, HDR activity is typically restricted to the S/G2 phase of the cell cycle, which reduces the efficiency of targeted genome engineering [24,25,26]. Because HDR has a lower frequency of DSB repair compared to NHEJ [27,28], a NHEJ-mediated targeted integration pathway, known as the homology-independent targeted integration (HITI) strategy, has been developed. HITI shows potential for knocking foreign genes into target gene loci in both dividing and non-dividing cells [29]. Previous studies have demonstrated that the genome editing efficiency of HITI in cell lines and tissues can reach 40% and 20%, respectively, while that of HDR is less than 10% [29]. This highlights HITI as a promising technique for engineering the genomes of non-dividing cells at the terminal stage of differentiation, such as most cells in the inner ear.

In this article, we used the NHEJ-mediated HITI strategy to determine the applicability of CRISPR therapy at this site. Specifically, we used HITI to insert a wild-type genomic sequence near the *SLC26A4* c.919-2A>G variant into human induced pluripotent stem cells (iPSCs) [21] and HEK293T cells to explore the potential of HITI-mediated gene correction therapies in this genomic region.

## 2. Results

### 2.1. The Surveyor Assay Results

**Figure 1:**
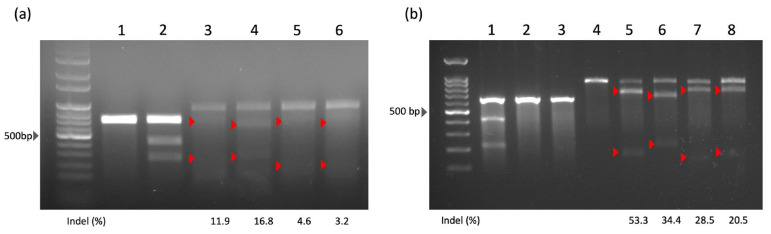
Surveyor assay results in iPSCs and HEK293T cells. (**a**) The Surveyor assay was per-formed to analyze the efficiency of designed sgRNAs in iPSCs. The lane assignments are as follows: 1. negative control; 2. positive control; 3. Cas9/sgRNA 1; 4. Cas9/sgRNA 2; 5. Cas9/sgRNA 3; 6. Cas9/sgRNA 4. (**b**) The Surveyor assay was also performed in HEK293T cells using the same set of designed sgRNAs. The lane assignments are as follows: 1. positive control; 2, 3. negative controls from G/C plasmids; 4. Cas9 mock plasmid; 5. Cas9/sgRNA1; 6. Cas9/sgRNA2; 7. Cas9/sgRNA3; 8. Cas9/sgRNA4. The red triangles indicate the cleavage products generated by the Surveyor assay.

**Figure A1:**
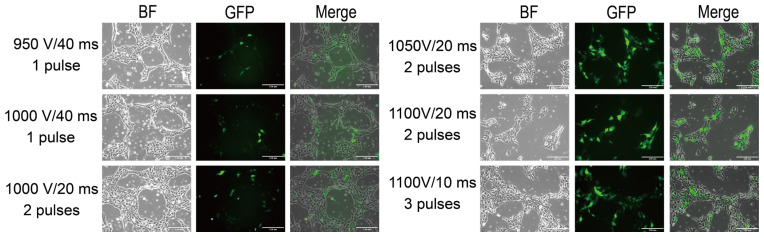
Optimization of Electroporation Efficiency in iPSCs. Various pulse voltages, pulse widths, and pulse numbers were tested to evaluate transfection efficiency based on subsequent fluorescence expression. Surviving iPSCs were subjected to puromycin selection to determine the most suitable electroporation conditions. BF: bright field.

According to our design, PCR amplification is expected to yield 933 pb products. Depending on the specific sgRNA used (Table 1), the Surveyor assay results will produce fragments of varying sizes as follows: sgRNA1: 226 + 707 bp; sgRNA2: 279 + 654 bp; sgRNA3: 190 + 743 bp; sgRNA4: 203 + 730 bp (Figure 1). Unlike HEK293T cells, iPSCs are more challenging to transfect with the HITI plasmid. Therefore, we employed electroporation and tested various conditions to identify the optimal efficiency and the highest cell survival rate (Figure A1). Since the sgRNA efficiency observed in iPSCs was significantly lower than that in HEK293T cells, we conducted the subsequent pipeline of HITI experiments using only HEK293T cells.

In HEK293T cells, sgRNA1 showed the highest targeting efficiency (53.3%), followed by sgRNA2 (34.4%), sgRNA3 (28.5%), and sgRNA4 (20.5%). However, previous studies [30,31] have shown that the polypyrimidine tract is an important cis-acting sequence element that directs intron removal during mRNA splicing. The sgRNA1 sequence contains abundant pyrimidines, which may be a critical region for intron splicing. In addition, there is increasing evidence that exon elements (targeted by sgRNA2) may play an important role in splice site selection [31,32]. Since it was unclear whether integration would have negative effects on the pre-mRNA splicing process in the targeted variant of our interest, we excluded sgRNA1 and sgRNA2 to avoid any unexpected results. As a result, sgRNA3 was selected as the ideal candidate for further evaluation of HITI performance.

### 2.2. Statistics of HITI Editing Efficiency

**Figure 2:**
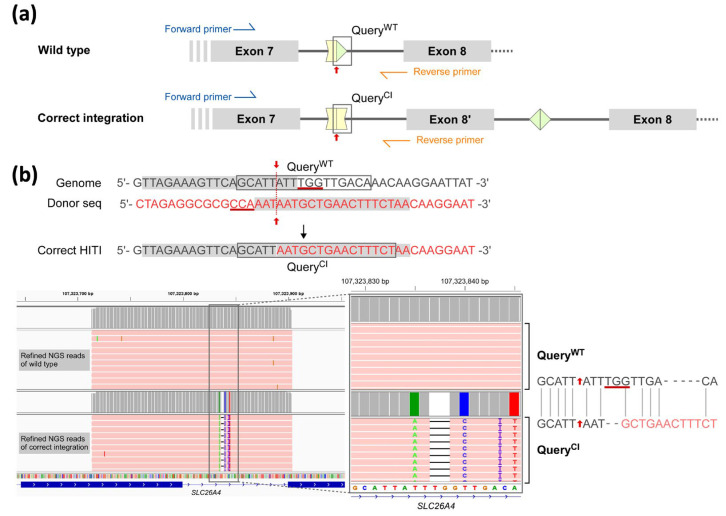
HITI-mediated correct integration with distinct query and the corresponding NGS data. (**a**) Schematic of the wild-type and correct integration genomes, as well as the forward/reverse primers (blue/orange arrows) and distinguished queries (Query^WT^ vs. Query^CI^ framed by black lines). Red arrow: Cas9 cutting site. (**b**) The sequences before and after correct integration (Query^WT^ vs. Query^CI^ in framed region) and the refined NGS reads of SLC-2 mapped onto *SLC26A4* genomic region that harbors Query^WT^ or Query^CI^ (framed region in lower-left panel), accompanied by the zoom-in view and pairwise alignment for the Query^WT^ and Query^CI^ segment (right panel). The Cas9/gRNA target sequence is shown in grey. The PAM sequence is underlined.

Three experimental samples (SLC-1 to -3, detailed in Section 4.4) were evaluated to determine their editing efficiencies using the next-generation sequencing (NGS) approach. As shown in Figure 2a, amplicons containing partial exon 7, intron 7, and partial exon 8 (chr7:107,323,714–107,323,903) from either the wild type (top panel) or the correct integration counterpart (bottom panel with head-to-head joining of the 5′ junction segments across the CRISPR cutting site) were generated using the same PCR primers for deep NGS sequencing. The two discrepant segments Query^WT^ and Query^CI^ (frame in black line in Figure 2a,b), derived from amplicons of each sample across the CRISPR cutting sites, were selected as distinct markers of wild-type and correct integration, respectively, to detect each set of distinct NGS reads. The results of the NGS assays are summarized in Table 2. By filtering out noise reads that mapped beyond the amplified target region, approximately 97–99% of the raw NGS reads were retained as refined reads. For all refined reads, those containing Query^WT^ segments and various degrees of editing events in the samples, including correct integration (i.e., containing Query^CI^) or small deletion/insertion/indel adjacent to the cutting site, are summarized in Table 2. Notably, SLC-2 had a lower proportion of Query^WT^ segments (~91%) and more editing events than the other two, including 27 reads with correct integration (27/18 181, 0.15%) containing Query^CI^ in the NGS assay (Figure 2b). These results indicate that the HITI strategy, which transfected both sgRNA and donor plasmids, successfully introduced the designed knock-in sequence at the junction of the CRISPR editing sites.

## 3. Discussion

Despite their clinical significance, the pathogenic mechanisms underlying *SLC26A4* variants remain largely unexplored, leading to a lack of effective treatments. Potential therapeutic solutions for *SLC26A4* variants include gene replacement (or augmentation) and genome editing to correct specific variants. In 2019, Kim et al. [33] demonstrated the potential of gene replacement therapy for pendrin-related hearing loss by injecting an adeno-associated virus containing *SLC26A4* cDNA into the inner ear of *SLC26A4* knockout mice. Later, Takeda et al. [34] demonstrated that transuterine gene transfer of *SLC26A4* cDNA into the otocysts of *SLC26A4*-deficient mice could restore hearing and vestibular functions. However, these replacement approaches, by providing exogenous coding sequences, may result in complications arising from overexpression or ectopic expression of the wild-type gene in treated cells [19,20,21]. In addition, gene replacement therapies have limited durability and may require repeated administration, increasing the likelihood of side effects such as immunogenicity [22].

In contrast, genome editing with the CRISPR/Cas9 system offers a potential one-time treatment strategy to efficiently and permanently correct pathogenic variants. Recently, several derivative techniques have been developed to improve the performance of the CRISPR/Cas9 system, such as base editing and prime editing [35]. Base editing can directly modify a single nucleotide without a template, while prime editing can provide more possible sites of action to increase the editing efficiency [36]. Despite these advances, the long-term efficacy of these genome editing approaches in vivo is controversial [37]. In addition, most somatic cells are non-dividing, which limits the development of therapeutic strategies utilizing the aforementioned CRISPR/Cas9 techniques that require a precise HDR pathway [24,38,39,40]. This may be particularly relevant for the treatment of HHI, as most cells in the inner ear are in the terminal stage of differentiation and are non-dividing. The collaborative base editing experiments conducted with the David Liu laboratory at Harvard University further exemplify the low efficiency of editing at this locus. Notably, more than ten types of off-target edits were detected within the 40-nucleotide region flanking the variant site, while only 0.22% of reads exhibited the desired correction. It is unclear whether the low correction rates resulted from the genomic sequence, location, or the CRISPR/Cas9 strategy itself.

Recently, a unique CRISPR/Cas9-based HITI strategy was developed to enable targeted gene insertion in non-dividing cells both in vitro and in vivo, thus showing great translational potential for inner ear therapeutics [29]. In this study, we investigated the editing efficiency of HITI in human cells harboring *SLC26A4* c.919-2A>G, the most common *SLC26A4* variant in East Asian populations. The aim of this study was to correct the *SLC26A4* transcript mis-splicing caused by the c.919-2A>G variant (chr7-107,323,898-A-G) by introducing the designed HITI donor sequence with the entire exon 8 and its flanking intronic segments (chr7:107,323,848–107,325,735, 1888bp) into the target region with the aim of restoring normal mRNA spliced transcript with non-skipped exon 8. To validate the efficiency of the HITI strategy, we used NGS to determine whether the donor sequence was successfully inserted into the expected locus. Although the HEK293T cells suggested a better sgRNA targeting efficiency than iPSCs, only 0.15% of the sequencing reads showed correct integration in the final performance of HITI.

To our knowledge, this study was among the first in the literature to apply HITI to correct pathogenic HHI variants. Despite the low efficiency, the presence of the Query^CI^ sequence (Figure 2b) indicated that HITI-mediated genome editing had occurred, providing proof of concept. Several factors likely contributed to the low editing efficiency. First, based on our experience with other CRISPR/Cas9 assays, the T-rich sequence near the c.919-2 locus may affect the sgRNA binding efficiency. Second, HITI-mediated genome editing requires the donor sequence to be in a specific forward/reverse orientation, which may require multiple CRISPR cutting steps and reduce efficiency. Third, to be compatible with the adeno-associated virus system, our CRISPR/Cas9 system was cloned separately into the two-plasmid system; therefore, only double-transfected cells had a chance to be edited, which reduced the yield of successful transfection.

Given that the observed editing rate was extremely low, we anticipated that the actual efficiency in in vivo settings, where additional delivery barriers exist, would be further reduced to a level without practical therapeutic relevance. Therefore, we decided not to pursue downstream functional assays such as splicing rescue or protein expression analysis, and instead concluded that the HITI approach, at least in its current design, is not suitable for targeting the *SLC26A4* c.919-2A>G variant.

Although HITI did not yield satisfactory results at the target site investigated in our study, it has already been demonstrated to achieve high editing efficiencies in certain tissues and cell types [29,41,42,43,44]. This fact further suggests that factors beyond the HITI system itself—particularly those related to the sequence context—may play a decisive role in determining editing success. Therefore, it is equally important to report both successful and unsuccessful experiences at different loci. We hope that our findings can serve as a valuable reference for researchers focused on both practical applications and technology development in this field.

In addition to the inherent challenges posed by the c.919-2A>G locus, we acknowledge that the choice of delivery method may significantly affect editing outcomes, particularly in hard-to-transfect cells like iPSCs. Although this study employed a plasmid-based approach, future strategies could explore the use of RNP electroporation or viral vectors such as AAV to improve transfection efficiency. Moreover, given the extremely low editing efficiency observed at this site, it may be beneficial to consider alternative strategies in both target selection and donor design. For example, shifting the target region further downstream of exon 8 may help avoid the restrictive sequence environment near the canonical splice site and offer more favorable sgRNA options. Additionally, although our donor construct was designed to comply with the AAV packaging size limit for potential clinical application, future studies not constrained by this requirement might consider using minicircle DNA as a donor format, which has been reported to enhance editing efficiency, particularly in non-dividing cells [29]. Taken together, these future directions may help overcome the intrinsic limitations at this locus and improve the feasibility of therapeutic genome editing for *SLC26A4*-related hearing loss.

The emergence of the HITI strategy provides a promising approach for genome editing in non-dividing inner ear cells during terminal differentiation. However, our findings suggest that the *SLC26A4* c.919-2A>G locus, due to its sequence constraints and editing inefficiency, may not be a suitable target for CRISPR/Cas9-based correction in its current configuration. Rather than pursuing further optimization at this site, our study emphasizes the importance of considering sequence context, donor design constraints, and delivery methods in developing future strategies. The experience gained through this work—particularly in dealing with difficult-to-edit genomic regions—may serve as a valuable reference for guiding the design of more effective approaches for other pathogenic variants associated with HHI. Our findings contribute meaningful insights to inform future research and help avoid trial-and-error approaches. Given the locus-specific complexity and genetic diversity of HHI, continued investigation into the translational potential of genome editing tools such as HITI remains warranted.

## 4. Materials and Methods

### 4.1. HITI Plasmid System

**Figure 3:**
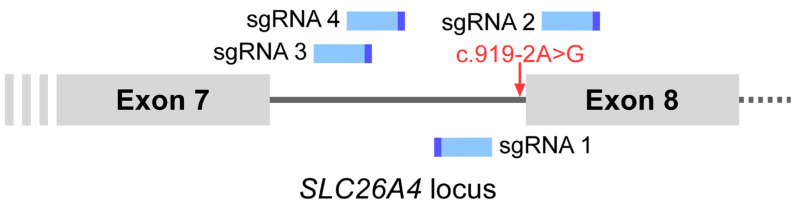
The candidate sgRNAs with putative targeting sites surrounding the *SLC26A4* c.919-2A>G variant. The sgRNA is shown with a 5′ protospacer (light blue) and a 3′ PAM motif (indigo).

The c.919-2A>G variant of *SLC26A4* (chr7-107,323,898-A-G) is located at the splice site of intron 7 and leads to aberrant splicing at the post-transcriptional level. Accordingly, we replaced the deleterious segment containing c.919-2A>G with a wild-type segment using the HITI strategy. CRISPR-Cas9 sgRNAs were designed using CRISPR design software (RGEN Cas-designer, Seoul, Republic of Korea, http://www.rgenome.net/). sgRNAs 1 and 2 were designed around the variant site, while sgRNAs 3 and 4 were designed in the middle of intron 7 (Figure 3). The sgRNA designs and predicted out-of-frame scores are listed in Table 1.

**Figure 4:**
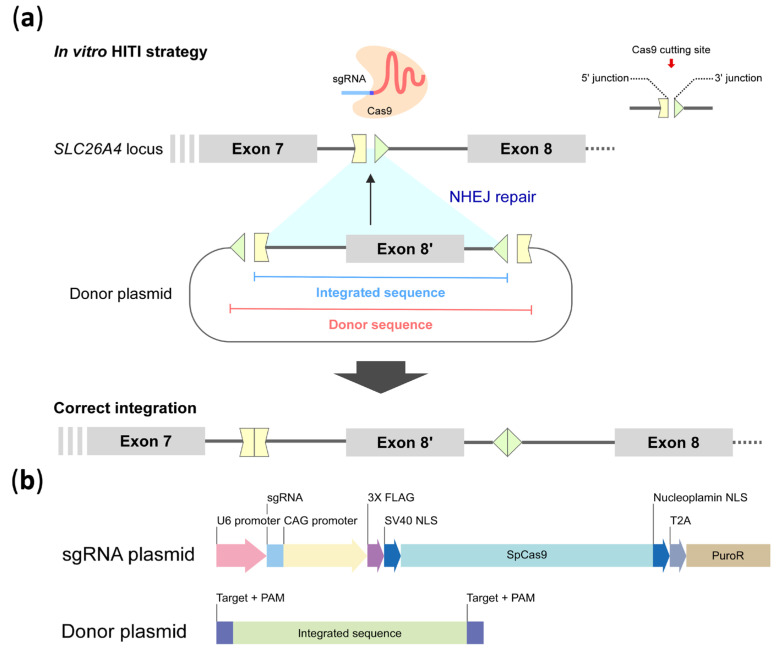
(**a**) Mechanism of the in vitro HITI strategy and correct integration of the CRISPR-mediated process. The integrated sequence, consisting of the genomic segment (exon 8′ and partial intron 7/8 colored in green) flanked by the reverse complement 5′ and 3′ junction segments, respectively, is integrated into the genome via non-homologous end-joining (NHEJ) repair to generate correct integration of the edited genome. (**b**) Plasmid system of sgRNA expression vectors (PX459) and donor plasmids for CRISPR experiment. CAG protomer: CMV early enhancer/chicken β actin protomer; NLS: nuclear localization signal; SpCas9: Streptococcus pyogenes Cas9 nuclease; T2A: self-cleaving peptides; PuroR: puromycin resistance gene.

Next, as shown in Figure 4a, the HITI donor sequence consisting of the wild-type genomic segment over partial intron 7, entire exon 8, and partial intron 8 (chr7:107,323,848–107,325,735, 1888 bp) of *SLC26A4* flanked by the reverse complement of the sgRNA sequence was constructed in the donor plasmid (middle panel). CRISPR homologous clipping would induce DSBs at 3 bp upstream of the PAM sites, allowing the sgRNA-targeted sequence to be split into 5′ and 3′ junction segments (top panel) in both the targeted genome and donor plasmid. The “integrated sequence” segment would be clipped from the donor plasmid and inserted into the junction site on the target genome via NHEJ-mediated repair (middle panel), resulting in the “correct integration” of the edited genome (bottom panel).

The expression vectors, containing each 20-bp target sequence subcloned into pSpCas9(BB)-2A-Puro (PX459) V2.0, were engineered as sgRNA expression vectors (a gift from Feng Zhang, Addgene plasmid # 62988; http://n2t.net/addgene:62988; RRID:Addgene_62988, Watertown, MA, USA) [45]. The targeting efficiency of sgRNA was examined in HEK293T cells.

Based on the results of the efficiency assay (mentioned in Section 2.1), the ideal sgRNA was cloned into the CRISPR/Cas9 plasmid. The PAM site (5′-TGG) in intron 7 was used as the target sequence of the guide RNA for the CRISPR experiment, along with its upstream 20 bp sequences (5′-TTAGAAAGTTCAGCATTATTTGG). The two-plasmid approach referred in a previous study [29] was conducted in our HITI strategy (Figure 4b). The donor sequence was obtained using a forward primer containing a BstBI restriction enzyme site and a reverse primer containing a MluI cutting site. The enhanced green fluorescent protein expression site and the sgRNA cassette were deleted, and the donor sequence was subcloned into the B1268-sgRNA vector to prepare the donor plasmid. The sgRNA plasmid and donor plasmid were used in subsequent cell experiments to assess the efficiency of HITI. A fluorescent UGM plasmid (pXL-T3-Neo-UGm-cHS4X, a gift from Dr. You-Tzung Chen), was used as a reporter to confirm transfection.

### 4.2. Cell Culture and Transfection

HEK293T cells (without the mutation site, a gift from Dr. You-Tzung Chen) were cultured to form a monolayer in Dulbecco’s modified Eagle’s medium (Thermo Fisher Scientific, Waltham, MA, USA) supplemented with 10% fetal bovine serum and incubated at 37 °C in a humidified 5% CO_2_ condition. Transfection of HEK293T cells was performed using DharmaFECT transfection reagents (Horizon Discovery, Waterbeach, UK). After 24 h of transfection, puromycin (4 μg/mL) was added to the culture medium, and genomic DNA was extracted after 48 h using Blood & Cell Culture DNA Kits (QAIGEN, Hilden, Germany).

iPSCs carrying the *SLC26A4* c.919-2A>G variant were generated from peripheral blood mononuclear cells obtained from a 9-year-old male (https://hpscreg.eu/cell-line/CGMHi001-A, accessed on 13 November 2018) [21]. The iPSCs were cultured in Stemflex medium (Thermo Fisher Scientific, Baltimore, MD, USA) supplemented with 10 μM Y-27632 (Sigma-Aldrich, St. Louis, MO, USA). Prior to cell seeding, culture surfaces were coated with 4% BD BioCoat Matrigel™ (BD Biosciences, San Jose, CA, USA) in DMEM/F12 medium (Thermo Fisher Scientific, Baltimore, MD, USA) for 30 min at 37 °C. Frozen iPSCs were thawed and seeded into Matrigel coated culture dishes for overnight culturing. When the cells reached approximately 70% confluence, the medium was replaced with Y-27632-free Stemflex medium. For plasmid transfection, iPSCs were detached from the culture dishes using accutase (Thermo Fisher Scientific, Baltimore, MD, USA), followed by washing with dPBS (Gibco/BRL, Grand Island, NY, USA) and resuspension with Stemflex medium containing 10 μM Y-27632. The cell concentration was adjusted to 1–1.5 × 10^6^ cells/mL. To determine the optimal transfection conditions, we first tested a GFP-expressing plasmid pZG12C03 (ZGENEBIO Co., Taipei, Taiwan) (see Appendix A). Transfection was performed using the Invitrogen™ Neon™ Transfection System (Thermo Fisher Scientific, Baltimore, MD, USA) under the following condition: 1050 V, 20 ms, and 2 pulses. After electroporation, cells were treated with 0.27 μg/mL puromycin (Sigma-Aldrich, St. Louis, MO, USA, USA) for drug selection. After 48 h, cells were harvested for DNA extraction.

### 4.3. Targeting Efficiency of sgRNAs and Selection

The targeting efficiencies of sgRNAs were assessed for HITI integration. The sgRNA and SpCas9 expression plasmids were transfected into iPSCs and HEK293T cells, followed by the Surveyor assay using a SURVEYOR Mutation Detection Kit (Integrated DNA Technologies, Coralville, IA, USA). Briefly, genomic DNA (0.5 μg) was mixed with 2X GoTaq Green Master Mix (Promega, Madison, WI, USA) and 0.5 μM of primers (forward: 5′-TAGACGCTGGTTGAGATTTT-3′; reverse: 5′-TCGGCTGTTTTCATTATCCT-3′). The following conditions were used for PCR amplification: initial denaturation at 94 °C for 5 min, followed by 94 °C for 30 s, 55 °C for 60 s, and 72 °C for 30 s for 35 cycles. After purification with a QIAquick Gel Extraction Kit (QIAGEN, Hilden, Germany), PCR products were hybridized by heating and cooling the mixture to generate hetero- and homoduplexes, which were then treated with Surveyor nuclease at 37 °C. After resolving the reaction products in an agarose gel electrophoresis, the relative amounts of DNA were quantified using an UVP imaging system (UVP BioSpectrum^®^ 810 Imaging System, Thermo Fisher Scientific). Indels were calculated using the following formula, where a represents the intensity of the undigested fragment, while b and c denote the intensities of the cleavage products.

$$Indel\;\left(\%\right)=100\times \left(1-\sqrt{\frac{b+c}{a+b+c}}\right)$$

### 4.4. HITI

Experiments were divided into three groups: SLC-1 (donor plasmid only [1.5 μg DNA]), SLC-2: (donor plasmid [1.5 μg DNA] + sgRNA plasmid [0.5 μg DNA]), and SLC-3 (sgRNA plasmid [0.5 μg DNA] + UGM plasmid [1.5 μg DNA]). HEK293T cells were grown to 40% confluence in six-well plates. The three sets of above plasmids were transfected into each well using DharmaFECT transfection reagents (Horizon Discovery, Waterbeach, UK). Cells were harvested 72 h after transfection, and genomic DNA was extracted for NGS analysis.

### 4.5. Amplicon-Based NGS and Data Processing of Reads

High-throughput NGS assays were conducted to assess the performance of HITI approach. Cell lysate from each sample (SLC-1 to -3) was collected for targeted amplification using PCR primers (forward: 5′-CCATTGTCGTCTGTATGGCA; reverse: 5′-TCGTCTGAAATAAAACAAAAGATGT). All PCR products (~190 bp) were used in NGS experiments (pair-end 250 bp reads, HiSeq, Illumina, San Diego, CA, USA) to assess the degree of correct integration within the targeted genomic region (chr7: 107,323,714–107,323,903). The reads were mapped to the human genome (hg19 version) using BWA-MEM (v.0.7.17) [46], followed by mapping correction and base quality score recalibration using Picard (v. 1.134, Broad Institute, MA, USA http://broadinstitute.github.io/picard) and the GATK toolkit (v3.8.1.0) [47] to obtain the final alignment of the NGS reads. The Integrative Genomics Viewer platform [48] was used to display the reference-based in-silico alignment of the NGS reads, and Samtools (v.1.15.1) [49] was used for format conversion of the BAM/SAM files. BAMQL software [50] was used to remove noise reads outside the targeted mapping region and to retrieve the reads based on the queries of interest. The queries “5′-GCATTATTTGGTTGACA” and “5′-GCATTAATGCTGAACTTTCT”, defined as the markers of wild-type (Query^WT^) and correction integration (Query^CI^), respectively, were used to distinguish the NGS reads for assessing the performance of the HITI approach.

## Figures and Tables

**Table 1:** sgRNA designs and out-of-frame score prediction (RGEN Cas-designer).

No.	sgRNA and PAM Sequence	Out-of-Frame Scores
sgRNA1	AAAGATGTTAAAAACTCCAT TGG	53.9
sgRNA2	ATTGCTACTGCCATTTCATA TGG	69.8
sgRNA3	TTAGAAAGTTCAGCATTATT TGG	68.4
sgRNA4	CATTATTTGGTTGACAAACA AGG	70.5

**Table 2:** Summary of NGS reads derived from amplicons of HITI experiment.

Name	SLC-1 *	SLC-2 *	SLC-3 *
Raw reads	17,435	18,381	22,062
Refined reads	17,184	18,181	21,755
Refined reads with Query^WT^	16,466 (95.82%)	16,561 (91.09%)	20,688 (95.10%)
Refined reads with Query^CI^	0	27 (0.15%)	0
Refined reads with deletion	14 (0.08%)	193 (1.06%)	19 (0.09%)
Refined reads with insertion	0	434 (2.39%)	0
Refined reads with indel	0	3 (0.02%)	1 (<0.01%)

* SLC-1: donor plasmid only; SLC-2: donor plasmid + sgRNA plasmid; SLC-3: sgRNA plasmid + UGM.

## Data Availability

The data supporting the findings of this research are available from the corresponding author, Yen-Hui Chan and Yen-Fu Cheng, upon request.
